# Comparative microRNA-seq Analysis Depicts Candidate miRNAs Involved in Skin Color Differentiation in Red Tilapia

**DOI:** 10.3390/ijms19041209

**Published:** 2018-04-16

**Authors:** Lanmei Wang, Wenbin Zhu, Zaijie Dong, Feibiao Song, Juanjuan Dong, Jianjun Fu

**Affiliations:** 1 Freshwater Fisheries Research Centre of Chinese Academy of Fishery Sciences, Key Laboratory of Freshwater Fisheries and Germplasm Resources Utilization, Ministry of Agriculture, Wuxi 214081, China; wanglm@ffrc.cn (L.W.); zhuwb@ffrc.cn (W.Z.); fujj@ffrc.cn (J.F.); 2 Wuxi Fisheries College, Nanjing Agricultural University, Wuxi 214081, China; songfb1014@126.com (F.S.); m18114862625@163.com (J.D.)

**Keywords:** miR-seq, skin color, red tilapia, pigment, comparative analysis

## Abstract

Differentiation and variation in body color has been a growing limitation to the commercial value of red tilapia. Limited microRNA (miRNA) information is available on skin color differentiation and variation in fish so far. In this study, a high-throughput Illumina sequencing of sRNAs was conducted on three color varieties of red tilapia and 81,394,491 raw reads were generated. A total of 158 differentially expressed miRNAs (|log_2_(fold change)| ≥ 1 and *q*-value ≤ 0.001) were identified. Target prediction and functional analysis of color-related miRNAs showed that a variety of putative target genes—including *slc7a11*, *mc1r* and *asip*—played potential roles in pigmentation. Moreover; the miRNA-mRNA regulatory network was illustrated to elucidate the pigmentation differentiation, in which miR-138-5p and miR-722 were predicted to play important roles in regulating the pigmentation process. These results advance our understanding of the molecular mechanisms of skin pigmentation differentiation in red tilapia.

## 1. Introduction

Tilapia (genus *Oreochromis*), which originated in Africa, is one of the most important food fishes in the world. Due to its fast growth and high adaptability to complex cultural systems, tilapia is widely accepted and has emerged as the second most produced fish in aquaculture around the world [1,2]. There exist various kinds of tilapia species, including Nile tilapia (*O. niloticus*), blue tilapia (*O. aureus*), mutant reddish-orange Mozambique tilapia (*O. mossambicus*), red tilapia (*Oreochromis* sp. red tilapia) and others originating from a multi-crossbreeding strategy. Among those shown above, red tilapia, largely identifiable by its uniform red skin and bright pink peritoneum, is a very attractive commercial breed in certain markets.

Red tilapia usually display a variety of striking skin colors as a result of long-term natural selection, genetic regulation, feeding conditions, camouflage for threatening behaviors, mate-choice, and adaptation to low temperature, which play important roles in numerous biological processes [3,4,5,6,7]. The pigmentation differentiation in genetic breeding and skin color variation during the overwintering period are the main problems limiting the development of commercial red tilapia cultures. Coloration patterns including whole pink (WP), pink with scattered black spots (PB) and pink with scattered red spots (PR) have been found in the Malaysian red tilapia breeding population. The pigmentation differentiation is not reversible and skin color variation is reversible with the environmental temperature increasing [3,4,5,6,7]. Genetically, the formation of skin color has been proved to be a rather complicated bioprocess which mainly involves diverse pigments’ biosynthetic pathways determined by chromatophores or pigment cells associating with a series of cellular, nutritional, physiological and environmental factors [5,8]. It was reported that dozens of genes have been involved in the color pattern formation of zebrafish [9,10,11,12,13]. Several transcriptome analyses of non-model fish with various skin colors have been conducted to explore coloration-related genes and the genetic basis effectively [5,12,13].

MicroRNAs (miRNAs) are non-coding RNA molecules ranging from 21–25 nt which play important roles in regulating gene expression in biological processes. miRNAs have also been suggested to have a crucial role in the coloration of body skin. For instance, miR-137 affected body color pattern in mice [14], and loss of miR-8 decreased pigmentation of the dorsal abdomen in the fruit fly [15]. It has been reported that several miRNAs were related to the regulation of skin color in the common carp [16], and miR-429 seemed to be a potential regulator of pigmentation by targeting the 3’untransliated region (3′UTR) region of *foxd3* [17]. However, there are still few reports on the functions of miRNA regarding skin color differentiation.

In our previous study, an Illumina RNA-seq of transcriptome were conducted on three red tilapia varieties born with different colors (WP, PB and PR). We screened 148 differentially expressed genes (DEGs) and identified several key genes related to pigment synthesis, such as *tyr*, *tyrp1*, *pmel/silv*, *sox10*, *slc24a5*, *cbs* and *slc7a11* [5]. In this study, a high-throughput sequencing strategy was conducted to identify skin color-related miRNAs among these three types of coloration, and several differentially expressed miRNAs were validated by quantitative real-time polymerase chain reaction (qRT-PCR). Furthermore, putative target genes of differentially expressed miRNAs were predicted and a subsequent enrichment analysis of the Kyoto Encyclopedia of Genes and Genomes (KEGG) pathway was employed to gain insight into the function of miRNAs in the regulation of skin color in fish.

## 2. Results and Discussion

### 2.1. Signature of Sequencing Data in Red Tilapia.

**Figure 1:**
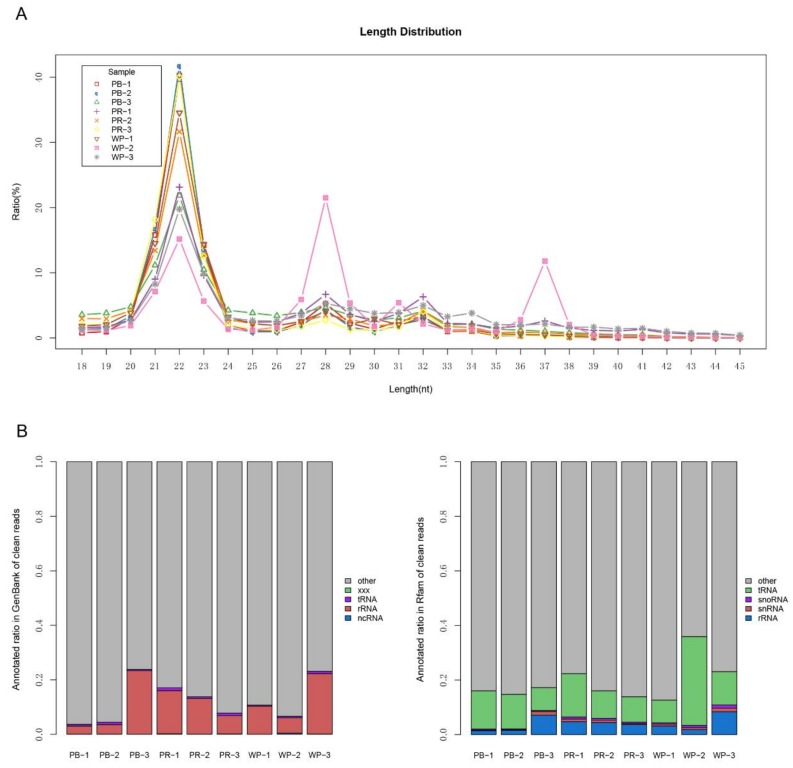
Overview of small RNA library sequencing. (**A**) Length distribution of small RNAs in red tilapia; (**B**) the clean reads were blasted against the GenBank and Rfam databases to annotate rRNA, tRNA, snRNA or snoRNA.

To obtain the miRNA expression signature related to red tilapia skin colors, we constructed small RNA sequencing libraries from 9 sample pools isolated from the skins of adult red tilapia with three different colors: WP, PB and PR (Supplementary Figure S1, triplicate for each color). An average 9,872,682 raw reads, corresponding to 9,043,832 clean reads after removing reads containing adaptor sequences and low-quality reads (Table 1), were obtained from each library. We summarized the length distribution of all clean reads and found that 8 samples, except one sample in the WP group, showed a peak of miRNA of typical length at 22 nt (Figure 1A). Approximately 53.32% to 68.73% clean reads were matched to the Nile tilapia genome (Supplementary Table S1). All clean reads were used for functional annotation (Figure 1B) and the remnant sRNA reads with rRNA removed were used for searching published mature miRNAs. The unannotated sRNA reads were processed to predict novel miRNAs using MIREAP software (http://sourceforge.net/projects/mireap). A total of 275 miRNAs were identified to be the same or similar to the mature miRNAs (known), and 205 miRNAs were predicted to be novel miRNAs with hairpin structures (novel, Supplementary Table S2).

### 2.2. MicroRNAs (miRNAs) Show Differential Expression in Tilapia with Different Skin Colors

**Figure 2:**
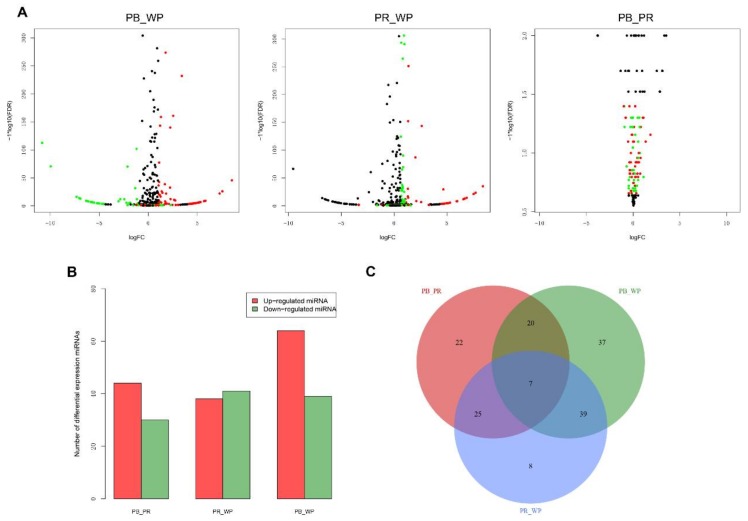
Differentially expressed miRNAs (DEMs) in three pairwise comparison groups. (**A**) The volcano plots of three pairwise comparisons. Dark, red, green dots represent non-significant, up-regulated and down-regulated miRNA genes, respectively; (**B**) red and blue indicate the number of up- and down-regulated miRNAs, respectively; (**C**) Venn diagram of differentially expressed miRNAs.

**Figure 3:**
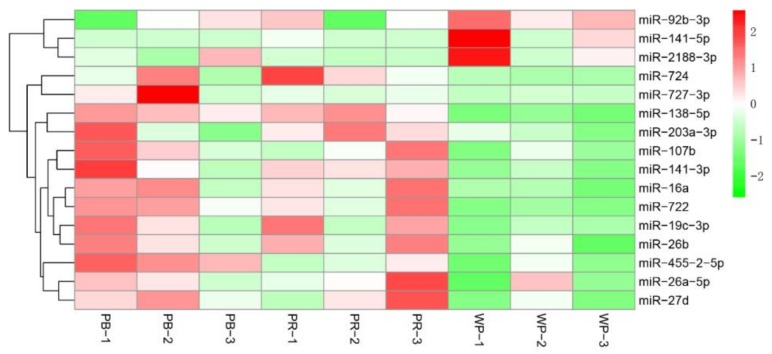
The expression pattern of 16 differentially expressed miRNAs (DEMs) related to pigmentation process. From green to red, the expression value ranged from low to high expression.

**Figure 4:**
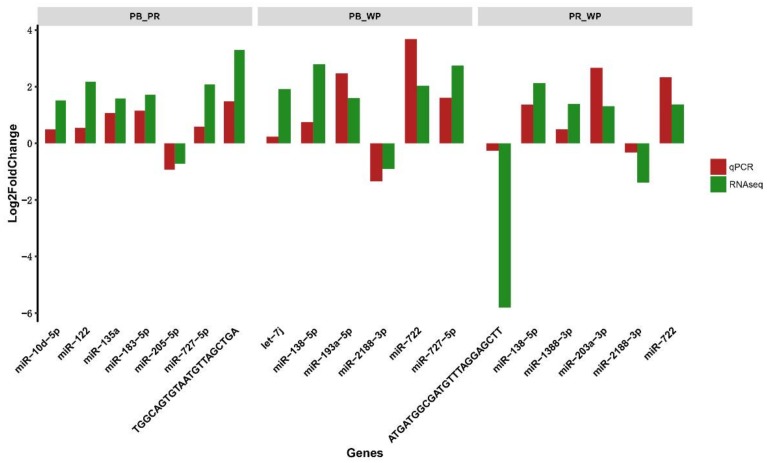
Comparison of relative expression levels of miRNAs between quantitative real-time polymerase chain reaction (RT-qPCR) and Illumina sequencing in three comparative groups of red tilapia.

With the criteria of |log_2_(Fold change)|  ≥  1 and *q*-value  ≤  0.001, we identified 103 significant differentially expressed miRNAs (DEMs) in PB (including 64 up-regulated and 39 down-regulated miRNAs) compared with WP (Figure 2A); 79 significant DEMs in PR (containing 38 up-regulated and 41 down-regulated miRNAs) compared with WP; and 74 significant DEMs in PB (containing 44 up-regulated and 30 down-regulated miRNAs) compared with PR (Figure 2B; Supplementary Table S3). Among these miRNAs, 158 miRNAs including 7 overlapped DEMs were identified (Figure 2C). Moreover, 46 miRNAs (including 16 known and 30 novel DEMs) were DEMs in the PB and PR groups compared with the WP group (Figure 2C). The heatmap of these 16 known DEMs indicated that most of them, except down-regulated miR-92b-3p, miR-141-5p, miR-2188-3p, were up-regulated in PB and PR skins compared with WP skins (Figure 3). We then randomly selected 15 DEMs (including 3 down-regulated and 12 up-regulated miRNAs) and validated the expression patterns using qRT-PCR. The results showed that the qRT-PCR expression patterns were absolutely in agreement with those from RNA-seq analysis (Figure 4), indicating the RNA-seq data was reliable.

### 2.3. Target Prediction for Differentially Expressed miRNAs (DEMs)

**Figure 5:**
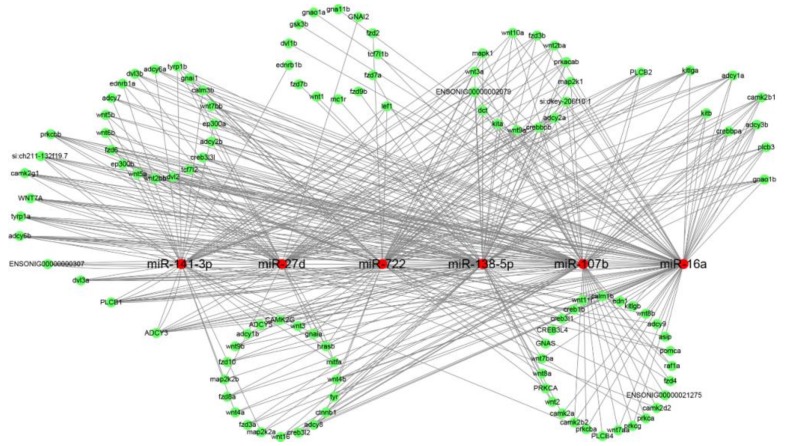
Network of putative interactions between miRNAs and their target genes related to the pigmentation process. The regulation network of miRNAs and their target genes was illustrated by Cytoscape. Red and green circles represent miRNAs and target genes, respectively.

Target prediction analysis of DEMs was conducted using the RNAhybrid, miRanda and targetscan algorithms. Results showed that 103, 79 and 73 DEMs corresponded to 11,966, 11,728, and 11,524 genes for three comparison. For example, miR-722 targeted 69 genes, including the wnt family genes (*wnt4*, *wnt5*) and tyrosinase-related protein family genes (*tyrp1*, *tyrp2*); miR-138-5p targeted 81 genes, including melanocortin 1 receptor (*mc1r*), transcription factor 7-like 2 (*tcf7l2*) and *dct*; microphthalmia-associated transcription factor (*mitf*) was a target of miR-141-3p, miR-27d, miR-26a-5p, and miR-455-2-5p (Supplementary Table S4). *Mitf* and *mc1r* indirectly regulate the melanogenesis [18,19], while some genes, including *tyr*, *dct*, *tyrp1*, have been reported to participate into melanogenesis and melanin synthesis, as illustrated in our previous study [5]. A regulatory network between these 6 DEMs and their targets was illustrated and showed their target genes were regulated by different miRNAs (Figure 5). These facts suggested a complicated regulatory network between miRNAs and their targets and complicated roles of these DEMs in pigmentation differentiation.

Previous observations have suggested that miR-107b was differentially expressed in the brain tissue of rainbow trout and found to target *sox6* [20], which might act as a transcription factor indirectly regulating embryonic development and melanin biosynthesis by modulating *sox10* in B16 melanoma cells [21]. The melanocortin system exerted its multiple functions via G protein-coupled receptors (GPCRs) [22]. In non-small cell lung cancer cells, GPR124, one of the GPCRs, was proved to be a direct target of miR-138-5p and its expression was suppressed by miR-138-5p [23]. Moreover, miR-141-3p was shown to indirectly interact with the GPCR signaling pathway via its targets [24]. MiR-27d, miR-16a and miR-722 had been identified as playing crucial roles in sex differentiation of tilapia, the cell cycle of whitefish liver cells, and immune system development and response, respectively [25,26,27,28]. However, their specific functions in pigmentation in red tilapia were poorly reported. In this present study, the fact that these four DEMs, miR-107b, miR-138-5p, miR-141-3p and miR-27d, were up-regulated in fish with PB and PR skins compared with fish with WP skins suggested that they played important roles in pigmentation in red tilapia.

**Figure 6:**
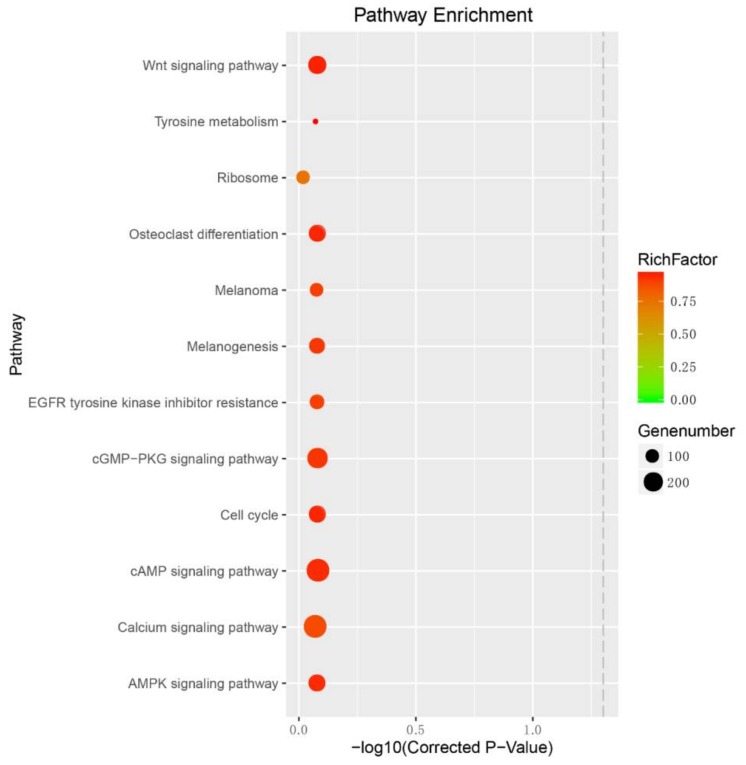
Pigmentation-related Kyotoo Encyclopedia of Genes and Genomes (KEGG) pathway of predicted target genes of Differentially Expressed miRNAs (DEMs) in red tilapia. Gene number—number of target genes in each pathway. Rich factor—the ratio of the number of target genes divided by the number of all the genes in each pathway.

To predict the metabolism pathway of the target genes of these 16 interested DEMs between fishes with PB and PR skins, KEGG pathway analysis was conducted on their target genes. Enrichment analysis showed that their target genes were classified into several pigmentation-related pathways, including the Wnt signaling pathway, the MAPK (mitogen-activated protein kinase) signaling pathway, and melanogenesis (Figure 6; Supplementary Table S5). Among the targets, *mc1r*, the most important regulator of melanogenesis, positively regulated cyclic AMP (cAMP) response-element binding protein (*creb*) and downstream *mitf* to increase melanin synthesis [29]; *wnt4* was also reported to be maintained at higher levels in normal peripheral retinal pigment epithelium cells in dark agouti rats [30]. In normal melanocytes, the activation of Mc1r-cAMP-Mift pigmentation signaling cascade has been proved to induce the production of transforming growth factor (TGF)-β1 in transformed melanocytes in human melanoma cells and play crucial roles in ultraviolet (UV) light exposure-induced skin pigmentation [31,32]. Moreover, the inactivation of the Mc1r-cAMP-Mift signaling cascade was essential for sesamol-decreased pigment formation and melanogenesis in melanocyte cells and zebrafish [33].

Recently, a transcriptome analysis showed *wnt4* related to melanogenesis in common buzzards with different color [34]. The gallic acid-induced inhibition of melanogenesis and hypopigmentation in B16F10 cells was associated with the activation of Akt, MEK/ERK (extracellular regulated protein kinases), and Wnt/β-catenin signaling [35]. Also, *mift* was reported to be required for β-catenin-induced melanoma growth [19]. These studies suggested that all these DEMs played crucial roles in pigmentation in red tilapia by modulating the related pathways via its target genes.

## 3. Methods and Materials

### 3.1. Ethics Statement

This study was approved by the Bioethical Committee of Freshwater Fisheries Research Center (FFRC) of Chinese Academy of Fishery Sciences (CAFS) (BC 2013863, 9/2013). The methods of all experiments were carried out in accordance with the Guide for the Care and Use of Experimental Animals of China.

### 3.2. Sample Collection

Red tilapias born with WP, PB and PR skin colors (53.1  ±  1.30 g, Supplementary Figure S1), were obtained from the Qiting Pilot Research Station (Yixing, Jiangsu, China), affiliated to the Freshwater Fisheries Research Center (FFRC), Chinese Academy of Fishery Sciences. Fishes were maintained in conical fibreglass tanks (water depth: 50 cm, volume: 256 L) in a flow-through water system during the acclimation and experimental periods. Water temperature was maintained at 27  ±  1 °C, pH  =  7–8, with dissolved oxygen (DO)  >  6 mg·L^−1^ and NH_4_-N  <  0.5 mg·L^−1^. Aeration was supplied 24 h per day and the photoperiod was 12D:12L.

Skin tissues were collected from 18 WP, 18 PB (pink, scattered 2/3 or above with black spots on all skin) and 18 PR (pink, scattered 2/3 or above with red spots of all skin) red tilapia individuals, respectively. All samples were immediately frozen in liquid nitrogen and then stored at −80 °C before RNA isolation. 

### 3.3. Small RNA Library Preparation and Sequencing

Total RNA was extracted from skin tissues using TRIZOL (Invitrogen, Carlsbad, CA, USA) according to the manufacturer’s protocol. Then genomic DNA was removed from RNA samples using DNase (New England Biolabs, Ipswich, MA, USA), and RNA purity was assessed using a Nanodrop2000 spectrophotometer (Thermo Fisher Scientific, BRIMS, Cambridge, MA, USA). An equal amount of total RNA from 6 individuals from each color group (WP, PB and PR red tilapia) was pooled, and 9 samples or RNA pools, including 54 individuals, were prepared accordingly. The purified RNA pools were ligated with Illumina 3′ and 5′ adapters (Illumina, San Diego, CA, USA) using T4 ligase (New England Biolabs, Ipswich, MA, USA). RNA was reverse-transcribed into the first strand cDNA using reverse transcriptase and amplified by PCR for 15 cycles using primers complementary to the adaptor sequences. Then, nucleotide fractions 140–150 bp in length were purified for Illumina sequencing library preparation. For sequencing, each library was loaded into one lane in the mode of 50 bp single-end Illunima Hiseq 2500 (Illumina, San Diego, CA, USA). 

### 3.4. Sequencing Analysis for miRNA Profiling

Quality control of all sequencing reads was conducted by FastQC software (http://www.bioinformatics.babraham.ac.uk/projects/fastqc/). We removed sequences in disorder, including poor quality reads, 3′ adaptor null reads, reads with 5′ adaptor contaminants, and reads shorter than 18 nt. The remaining sequences were used for mapping to the whole genome sequence of Nile tilapia [36], using the SOAP2 program (http://soap.genomics.org.cn) with a tolerance of 2 mismatches [37]. Then, rRNAs, tRNAs, snRNAs and snoRNAs in the matched sequences were filtered out by blasting against Rfam (http://rfam.sanger.ac.uk/) and National Center for Biotechnology Information (NCBI) GenBank (http://www.ncbi.nlm.nih.gov/genbank/) databases. After being classified into different categories, the remaining reads were aligned to the miRNA precursors in zebrafish in the miRBase version 21 database (http://www.mirbase.org/) to identify conserved miRNAs [38]. The unannotated sRNAs reads were conducted using MIREAP software (http://sourceforge.net/projects/mireap) to predict candidate novel miRNAs.

### 3.5. Data Normalization, Processing and Identification of DEMs

Sequencing data was normalized using the following formula: normalized expression = (actual miRNA sequencing readscount/total miRNAs reads count) × 1,000,000. Finally, DEGseq [39] was used to identify DEMs based on the miRNAs expression values by pairwise comparison. The cut-off criteria for DEMs were |log_2_(Fold change)| ≥ 1 and *q*-value ≤ 0.001.

### 3.6. Target Prediction for DEMs 

The target genes of DEMs were identified using the RNAhybrid [40], miRanda [41] and Targetscan 7.0 [42] with default settings. The genes supported by either two algorithms were considered as the targets of DEMs in this study. The target reference sequences were whole genome sequences of Nile tilapia.

### 3.7. Enrichment Analysis for DEM Targets

The KEGG pathway database informs people of how molecules or genes work [43]. In order to investigate the related biological function of DEM targets in the pigmentation, the pathways of biochemical and signal transduction significantly associated with the DEM targets were determined through the KEGG pathway (available online: http://www.kegg.jp/) analysis using the KOBAS server [44].

### 3.8. MiRNA-mRNA Regulatory Network Analysis

According to the regulatory relationships between key DEMs and their targets, the regulatory network containing key miRNAs and target mRNAs was constructed and visualized by Cytoscape software, a standard tool for integrated analysis and visualization of biological networks [45].

### 3.9. QRT-PCR Analysis

Total RNA was extracted as described above. For the reverse transcription of miRNAs, the Prime Script RT Reagent Kit (Takara Bio, Dalian, China) were used. QRT-PCR was performed on the ABI PRISM 7500 Real-time PCR System (Applied Biosystems, Foster City, CA, USA) using the 2X SYBR Green Master Mix reagent (Takara Bio, Dalian, China) following the below steps: initial denaturation at 95 °C for 5 min, followed by 95 °C for 15 s and 60 °C for 45 s for 40 cycles, and one cycle of 95 °C to 65 °C. All reactions were conducted in triplicate. The relative expression levels of the DEMs were measured in terms of threshold cycle value (Ct) and were normalized to 5S rRNA using the equation 2^−ΔΔ*C*t^. The miRNA specific primers (Supplementary Table S6) were synthesized by Genscript Inc. (Nanjing, China).

## 4. Conclusions

In this study, we screened the DEMs related to red tilapia pigmentation and predicted their target genes which might be associated with pigmentation process and melanogenesis by being involved in signaling pathways, including the Mc1r-cAMP-Mift signaling cascade, via controlling target genes. Moreover, the Wnt and MAPK signaling pathways were predicted to be associated with the pigment formation in red tilapia. Our findings provided insights into the mechanisms underlying miRNA-mediated pigmentation in red tilapia. Further experiments are needed to validate the exact roles and molecular mechanisms of miRNAs and their targets in pigment formation in red tilapia.

## Figures and Tables

**Table 1:** Summary of the sequencing data from red tilapia.

Item	Reads Count (Percent)								
	PB-1 *	PB-2	PB-3	PR-1 *	PR-2	PR-3	WP-1 *	WP-2	WP-3
Total reads	9745021	10172163	9597796	9138122	9086032	8568364	9696144	10741698	12108798
High-quality	9712465	10139260	9567374	9110612	9061737	8542050	9664568	10707005	12069765
	(100%)	(100%)	(100%)	(100%)	(100%)	(100%)	(100%)	(100%)	(100%)
3′adapter_null	48695	34324	47557	100967	39684	45828	19254	44297	139902
	(0.50%)	(0.34%)	(0.50%)	(1.11%)	(0.44%)	(0.54%)	(0.20%)	(0.41%)	(1.16%)
Insert null	2723	7882	2208	5979	11028	6166	11419	3320	11089
	(0.03%)	(0.08%)	(0.02%)	(0.07%)	(0.12%)	(0.07%)	(0.12%)	(0.03%)	(0.09%)
5′adapter_contaminants	24540	37898	24172	20256	22141	36854	14270	11819	9483
	(0.25%)	(0.37%)	(0.25%)	(0.22%)	(0.24%)	(0.43%)	(0.15%)	(0.11%)	(0.08%)
Smaller_than_18nt	245041	685335	1303589	587330	1111375	811448	683397	413050	555142
	(2.52%)	(6.76%)	(13.63%)	(6.45%)	(12.26%)	(9.50%)	(7.07%)	(3.86%)	(4.60%)
Poly A	82	87	52	153	64	118	71	127	129
	(<0.01%)	(<0.01%)	(<0.01%)	(<0.01%)	(<0.01%)	(<0.01%)	(<0.01%)	(<0.01%)	(<0.01%)
Clean reads	9391384	9373734	8189796	8395927	7877445	7641636	8936157	10234392	11354020
	(96.69%)	(92.45%)	(85.60%)	(92.16%)	(86.93%)	(89.46%)	(92.46%)	(95.59%)	(94.07%)

* Skin color varieties of red tilapia: WP—whole pink, PB—pink with scattered black spots, PR—pink with scattered red spots.

## Supplementary Materials

The following are available online at http://www.mdpi.com/1422-0067/19/4/1209/s1.
