# Supplementary figures and images for: Comparative microRNA-seq Analysis Depicts Candidate miRNAs Involved in Skin Color Differentiation in Red Tilapia
Source: Int J Mol Sci. 2018 Apr 16;19(4):1209. doi: 10.3390/ijms19041209 (PMC5979384; doi:10.3390/ijms19041209)

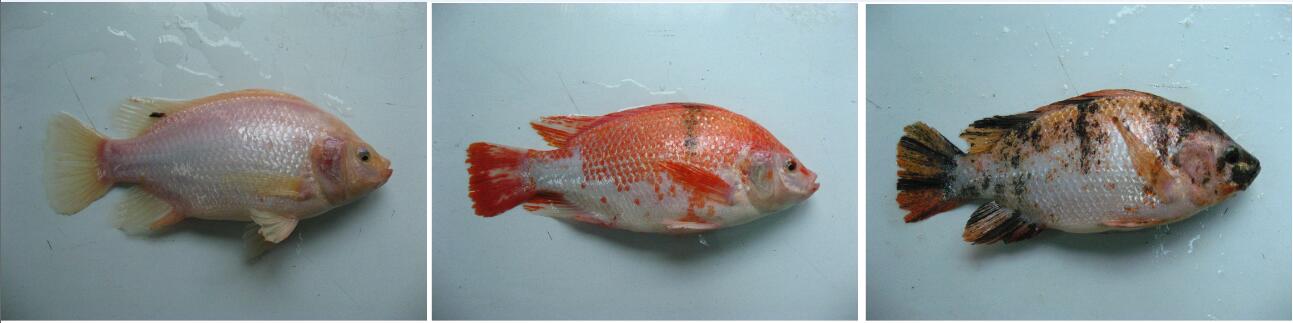

Supplement: Supplementary file 1 [file ijms-19-01209-s001.zip › ijms-285318-supplementary file/Supplementary Fig1.jpg]
